# Relationship Between Perceived Social Support and Physical Frailty Among Older Patients with Coronary Artery Disease: A Dual Mediation Model

**DOI:** 10.3390/jcm14051744

**Published:** 2025-03-05

**Authors:** Mihwa Won

**Affiliations:** Department of Nursing, Wonkwang University, Iksan 54538, Republic of Korea; mihwon7729@gmail.com; Tel.: +82-63-850-6045

**Keywords:** social support, health literacy, sedentary, frailty, older, coronary artery disease

## Abstract

**Background:** Physical frailty is common among older patients with coronary artery disease (CAD) and is influenced by perceived social support, health literacy, and a sedentary lifestyle. This study examined the dual mediating roles of health literacy and a sedentary lifestyle in the relationship between perceived social support and physical frailty in older patients with CAD. **Methods:** This cross-sectional study included data collected from 182 older patients with CAD at a general hospital in Korea between June 2022 and January 2023. Participants completed self-reported questionnaires measuring the main variables and demographic information. Clinical data were obtained from electronic medical records. **Results:** The mediation hypothesis was tested using a dual mediation model with the PROCESS macro (Model 6) and 95% bias-corrected bootstrap confidence intervals. Perceived social support indirectly influenced physical frailty through three pathways: health literacy alone, sedentary lifestyle alone, and a sequential process in which health literacy influenced sedentary lifestyle and ultimately physical frailty. **Conclusions:** This study identified a strong mediating effect of health literacy and a sedentary lifestyle on the relationship between perceived social support and physical frailty. A built environment of perceived social support among older patients with CAD improves health literacy, modifies sedentary lifestyles, and helps prevent physical frailty. Thus, healthcare professionals should evaluate the perceived social support, health literacy, and sedentary lifestyle of older patients when developing physical frailty prevention programs.

## 1. Introduction

Coronary artery disease (CAD) is a leading cause of morbidity and mortality in older adults [1]. Although CAD can occur in all age groups, its prevalence is significantly higher in older adults [2]. An estimated 315 million people are affected by CAD worldwide, with a prevalence of more than 70% in people aged 80 years and older, making it a major contributor to cardiovascular disease-related mortality [3,4]. Similarly, in South Korea, approximately 67.3% of patients diagnosed with CAD are between the ages of 60 and 80 [5]. Thus, the dual challenges of managing CAD and addressing the vulnerabilities associated with aging highlight the urgent need for targeted strategies to mitigate this growing public health issue.

Physical frailty refers to a decline in physiological functions associated with aging and is influenced by various factors, including disease, nutritional deficiencies, reduced physical activity, and stress [2]. It is a common comorbidity among older patients with CAD and significantly worsens disease outcomes [5,6]. The prevalence of physical frailty is significantly higher in older patients with CAD than in the general older population because the combined effects of cardiovascular disease and age-related deterioration exacerbate frailty [6,7,8]. For example, in older patients with CAD, physical frailty leads to reduced physical resilience, muscle strength, and endurance, thereby increasing the risk of adverse clinical outcomes, including reduced cardiopulmonary function, an increased risk of restenosis, heart failure remission, and mortality [6,9,10,11]. Therefore, assessing physical frailty among older patients with CAD in clinical settings is crucial as a proactive strategy because physical frailty worsens disease prognosis.

Perceived social support is the belief that accessible resources exist within one’s perceived social network [12]. Studies report that social support plays a crucial role in improving the physical and mental health of older adults by helping them solve problems, reducing stress, and encouraging a healthy lifestyle [13,14]. Social support has been demonstrated to promote physical activity and adherence in patients with cardiovascular conditions, such as stable CAD and heart failure [15,16]. Moreover, a cohort study found that older patients with limited social support had double the risk of frailty than those with robust social support [17].

Another significant determinant of frailty is health literacy, which is defined as a person’s capacity to access, comprehend, and apply information effectively [6]. A multi-center clinical trial reported that older patients with limited health literacy often have difficulty accessing health services, resulting in poor adherence to health behaviors [18]. Previous studies have demonstrated that older patients may experience worse physical frailty because they lack health literacy, such as the knowledge and confidence to effectively manage their condition [19,20].

In recent years, a sedentary lifestyle, characterized by prolonged periods of physical inactivity, has been recognized as a significant predictor of physical frailty [20]. The negative consequences of prolonged inactivity are well documented, with evidence suggesting that it accelerates the loss of muscle mass, reduces physical resilience, and contributes to the progression of frailty [7,21].

Despite the documented importance of perceived social support and its impact on physical frailty among older patients with CAD, evidence regarding this issue, particularly the mechanisms underlying this relationship in the Korean context, remains limited. Furthermore, given the cumulative nature of scientific research, it is essential to identify, appraise, and synthesize findings from individual studies on physical frailty among older patients with CAD. To address these gaps in the literature, the present study aims to examine the dual mediating effects of health literacy and a sedentary lifestyle on the relationship between perceived social support and physical frailty among older patients with CAD. Specifically, it examines the relationship between perceived social support and physical frailty while exploring the dual mediating roles of health literacy and a sedentary lifestyle in this association. The hypotheses of this study are as follows:

**Hypothesis 1:**  Does health literacy mediate the relationship between perceived social support and physical frailty?

**Hypothesis 2:**  Does a sedentary lifestyle mediate the relationship between perceived social support and physical frailty?

**Hypothesis 3:**  Do health literacy and a sedentary lifestyle jointly mediate the relationship between perceived social support and physical frailty as sequential mediators?

## 2. Materials and Methods

### 2.1. Study Design and Participants

The present study was designed as a cross-sectional study. The eligible population comprised older patients with CAD admitted to the cardiology ward of a general hospital in South Korea. The participants were recruited from June 2022 to January 2023 using convenience sampling.

The criteria for inclusion were as follows: (1) age of 65 years or older, (2) hospitalized patients undergoing percutaneous coronary intervention, (3) ability to read and answer the questionnaire independently or to fully understand its contents and provide answers when it is read to them, (4) and willingness to participate in the study. Participants were excluded if they were diagnosed with cerebrovascular disease, dementia, psychiatric disorders, end-stage cancer, or any other chronic diseases that could affect their cognitive function.

The required sample size was calculated using G*Power 3.1.9.2 software (Heinrich-Heine-Universität Düsseldorf, Düsseldorf, Germany), with the dual mediation analysis setting a two-tailed significance level of 0.05, mean effect size of 0.16, power of 0.95, and 12 predictor variables. This required a minimum sample size of 173 participants. Allowing a potential drop-out rate of 15%, 200 questionnaires were distributed. After the removal of 18 questionnaires that yielded insufficient responses, the final analysis included 182 questionnaires.

### 2.2. Questionnaire

#### 2.2.1. Demographic and Clinical Characteristics

The demographic and clinical characteristics of the participants were collected using an eight-item survey designed based on a comprehensive review of the literature [6,11,13]. These included variables such as age, sex, education level, living arrangements, duration since diagnosis, classification of angina severity according to the Canadian Cardiovascular Society (CCS) criteria, left ventricular ejection fraction (LVEF), and comorbidities. The CCS categorizes angina into four classes: occurring during strenuous activity (class I), moderate activity (class II), light activity (class III), or rest (class IV) [22].

#### 2.2.2. Perceived Social Support

The measurement of perceived social support was conducted utilizing the Health Care Climate Questionnaire [23]. It was developed to assess perceived social support from healthcare providers in clinical settings [23]. It consists of a seven-point Likert scale, ranging from 1 = “strongly disagree” to 4 = “neutral” and 7 = “strongly agree”. The total score ranges from 15 to 105, with higher scores indicating greater perceived social support. The reliability of the original scale was reported to be 0.89 at the time of its development [23] and was found to be 0.90 in the current study.

#### 2.2.3. Health Literacy

The Brief Health Literacy Screeners have been used to assess participants’ health literacy levels [24]. The present study used the Korean version of the Brief Health Literacy Screener [25], which has been validated in studies of patients with chronic diseases [26,27]. It has three components: confidence in completing medical documents, understanding written health information, and the ability to read and interpret hospital-related materials. This scale uses a rating scale ranging from 0 to 4, culminating in a maximum total score of 12, with higher values denoting an enhanced capacity to comprehend and utilize health-related information. In a previous study, its reliability was found to be 0.90 [26], whereas in the present study, its reliability was found to be 0.85.

#### 2.2.4. Sedentary Lifestyle

The Korean version of the Global Physical Activity Questionnaire was used to evaluate the sedentary lifestyle of the participants [28]. This scale, originally developed by the World Health Organization, was designed to assess physical activity in three domains—work, transport, and leisure—and sedentary lifestyle over the preceding seven days [29]. In the present study, a single item from the Korean version of the Global Physical Activity Questionnaire was used to measure the amount of time participants spent in a sedentary lifestyle [28]. The questionnaire covered various daily activities, including sitting at a desk, watching television, doing household chores, perceived socialization with friends, and commuting. The participants were asked to report the duration of these activities by indicating the time spent sitting or lying down during waking hours in hours and minutes, excluding sleep.

#### 2.2.5. Physical Frailty

The FRAIL scale was used to assess participants’ physical frailty status [30], with the Korean version of the scale validated for this study [31]. This scale assesses five domains: current level of fatigue, exercise tolerance, walking ability, number of medical conditions, and recent weight loss. The participants rated each domain on a scale from zero (indicating the best status) to five (indicating the worst status). Based on their scores, the participants were categorized into three groups: robust (score of zero), pre-frail (score of one to two), and frail (score of three–five). For the present study, participants were further divided into two categories: physical frailty (score of one or more) and non-physical frailty (score of zero) [11].

### 2.3. Procedure and Ethical Considerations

This study was approved by the Institutional Review Board of the researcher’s university. Prior to data collection, the researcher visited the target facilities, explained the purpose of the study and data collection methods to managers and practitioners, and obtained their permission to proceed with data collection. The study participants were recruited by posting flyers on ward bulletin boards. Two research assistants trained by the principal investigator in administering the questionnaire and data collection procedures screened electronic medical records to confirm eligibility.

Written consent was obtained from the participants, and data were collected using self-reported structured questionnaires and interviews. For participants who had difficulty reading and completing the questionnaire independently, the research assistants read the items aloud and recorded their responses. The questionnaire took approximately 20 min to complete, and a small gift was provided at the end of the survey. Participants’ privacy was protected by limiting the collection of identifiable personal information through the structured questionnaire.

### 2.4. Statistical Analysis

Data analysis was performed using IBM SPSS Statistics for Windows, version 27 (IBM Corp., Armonk, NY, USA). The demographic and clinical characteristics of the participants were analyzed using frequencies and percentages. The mean and standard deviation and minimum and maximum values of perceived social support, health literacy, sedentary lifestyle, and physical frailty were calculated. The normality of the data was assessed by examining skewness and kurtosis. Pearson’s correlation coefficient was used to examine the correlations between continuous variables (perceived social support, health literacy, and sedentary lifestyle). Point-biserial correlation was used to assess the relationship between continuous variables and the dichotomous variable (physical frailty).

The dual mediation effect was analyzed using the SPSS PROCESS macro (Andrew F. Hayes, Columbus, OH, USA) [32]. A 95% bootstrap confidence interval for the indirect effect estimate was calculated based on 10,000 bootstrap samples [32]. Based on Hays process model 6 [32], mediation analysis was used to examine the dual mediating roles of health literacy and sedentary lifestyle in the relationship between perceived social support and physical frailty. Indirect effects were assessed using point estimates and a 95% bias-corrected bootstrap confidence interval (CI). The effects were considered statistically significant if the CI did not include zero.

## 3. Results

### 3.1. Participants’ Characteristics

The demographic and clinical characteristics of the study participants are presented as follows: Among the 182 participants, the average age was 67.59 ± 7.49 years, with 116 males (63.7%) and 66 females (36.3%). The majority of participants had an education level below middle school (80 participants, 44.0%), followed by high school graduates (62 participants, 34.1%) and college graduates (40 participants, 22.0%). Additionally, 122 participants (67.0%) lived with their families.

The clinical characteristics include a mean duration since CAD diagnosis of 1.67 ± 3.56 years, with 74 of patients (40.7%) classified as having a CCI angina grade of class I. Regarding the LVEF, more than half of the participants (93 patients, 51.1%) had an LVEF of 41~49%, 58 patients (31.9%) had an LVEF of 50% or higher, and 31 patients (17.0%) had an LVEF of 40% or lower. Moreover, a significant proportion of participants had a clinical diagnosis of hypertension (69.2%) (Table 1).

### 3.2. Correlation Analysis of Main Variables

As shown in Table 2, the mean ± SD values for the perceived social support, health literacy, and sedentary lifestyle of older patients with CAD were 75.97 ± 14.62, 7.53 ± 2.99, and 8.08 ± 2.45 h/day, respectively. Among the total older patients with CAD, the prevalence of physical frailty was 127 (69.8%).

Participants’ perceived social support showed a statistically significant positive correlation with health literacy (*r =* 0.47, *p <* 0.001) and negative correlations with sedentary lifestyle (*r* = −0.54, *p* <0.001) and physical frailty (*rpb* = −0.60, *p <* 0.001). Also, health literacy was significantly negatively correlated with sedentary lifestyle (*r* = −0.49, *p <* 0.001) and physical frailty (*rpb* = −0.54, *p <* 0.001). Sedentary lifestyle demonstrated a significant positive correlation with physical frailty (*rpb* = 0.62, *p <* 0.001) (Table 2).

### 3.3. Dual Mediation Analysis Among Variables

**Figure 1:**
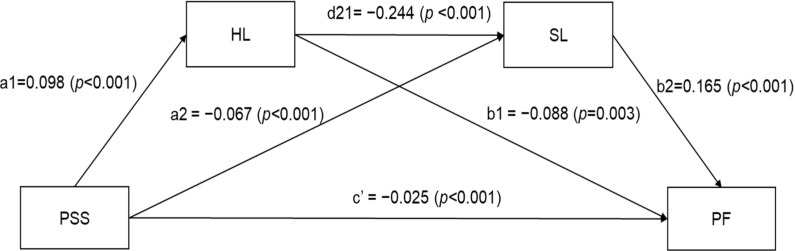
Dual mediation model of association between perceived social support and physical frailty through health literacy and sedentary lifestyle. PSS = perceived social support; HL = health literacy; SL = sedentary lifestyle; PF = physical frailty. a1 = direct effect of PSS on HL; a2 = direct effect of PSS on SL; b1 = direct effect of HL on PF; b2 = direct effect of SL on PF; d21 = direct effect of HL on SL; c = total effect of PSS on PF; c′ = direct effect of PSS on PF.

The results of the dual mediation analysis examining the effects of health literacy and sedentary lifestyle on the relationship between perceived social support and physical frailty are presented in Table 3 and Table 4. The dual mediation model with unstandardized path coefficient values is shown in Figure 1.

Initially, it was demonstrated that perceived social support exerted a statistically significant total effect on physical frailty among older patients with CAD (B = −0.050, t = −10.170, *p <* 0.001). Secondly, the direct effects of all paths were statistically significant. Namely, perceived social support was shown to have a positive association with health literacy through a direct effect (B = 0.098, *p <* 0.001) and a negative association with sedentary lifestyle through a direct effect (B = −0.067, *p <* 0.001).

Also, health literacy exhibited a negative association with sedentary lifestyle through a direct effect (B = −0.244, *p <* 0.001). Moreover, perceived social support (B = −0.025, *p <* 0.001), health literacy (B = −0.0888, *p* = 0.003), and sedentary lifestyle (B = 0.165, *p <* 0.001) were found to be negatively associated with physical frailty (Table 3, Figure 1).

Thirdly, the total indirect effects of perceived social support on physical frailty were found to be statistically significant, with a point estimate of −0.290 and 95% bootstrap CI (−0.034 to −0.014). The indirect effect of perceived social support on physical frailty through health literacy was statistically significant [indirect 1; point estimate = −0.1064, 95% bootstrap CI (−0.016 to −0.002)], and that through sedentary lifestyle was also statistically significant [indirect 2; point estimate = −0.135, 95% bootstrap CI (−0.019 to −0.005)].

The indirect effect of perceived social support on physical frailty through health literacy and sedentary lifestyle was statistically significant [indirect 3; point estimate = −0.0483, 95% bootstrap CI (−0.0076 to −0.0014)]. Consequently, perceived social support was found to be associated with health literacy, which, in turn, was linked to sedentary lifestyle and physical frailty (Table 4, Figure 1).

## 4. Discussion

The present study investigated the underlying mechanisms linking perceived social support with physical frailty, focusing on the mediating effects of health literacy and a sedentary lifestyle, in a convenience sample of older Korean patients with CAD hospitalized after percutaneous coronary intervention. The main findings of this study strongly support the hypotheses proposed in the dual mediation model. In particular, the results showed that health literacy and a sedentary lifestyle acted as sequential mediators in the relationship between perceived social support and physical frailty.

The limited exploration of dual mediators in the relationship between perceived social support and physical frailty in older patients with CAD makes a direct comparison of the results of the current study with those of previous research difficult. Nevertheless, the results are consistent with cross-sectional studies that have found significant associations between perceived social support and physical frailty, as well as between health literacy and physical frailty in older patients [17,19,20]. In addition, previous research has shown an association between a sedentary lifestyle and physical frailty in older patients with CAD [7].

The present study found a significant association between perceived social support and physical frailty among older patients with CAD, which is consistent with previous research [15]. Perceived social support has been consistently recognized as a protective factor against physical frailty, particularly among older adults with chronic conditions [33]. For example, Fang et al. reported that higher perceived social support was associated with lower physical frailty scores in China [15]. These results indicate that perceived social support by healthcare professionals is essential to improve disease management and alleviate physical frailty in older patients [34]. Thus, emotional and instrumental support provided by healthcare professionals can help reduce the stress associated with disease management in older patients with CAD and promote health-enhancing behaviors, thereby contributing to a reduction in physical frailty.

The statistically significant association between health literacy and physical frailty in older patients with CAD observed in the current study is consistent with previous research [35]. For example, a cross-sectional study of older Chinese patients with hypertension and diabetes found that lower health literacy was significantly associated with higher levels of physical frailty [36]. In addition, the significant association between a sedentary lifestyle and physical frailty in older patients with CAD is consistent with existing evidence [37]. In South Korea, a sedentary lifestyle is a common problem among older patients with CAD and is often influenced by cultural norms and environmental factors that discourage physical activity [38]. Therefore, the implementation of tailored interventions to address issues due to sedentary lifestyles is essential for reducing physical frailty and improving health outcomes in this population.

Interestingly, the concurrent mediating effects of health literacy and a sedentary lifestyle on the relationship between perceived social support and physical frailty in older patients with CAD have not been fully explored. Therefore, the results of this study contribute to the understanding of the role of health literacy and sedentary lifestyles in the association between perceived social support and physical frailty. The findings indicate that healthcare professionals play a crucial role in fostering an environment that supports health literacy improvement, promotes physical activity, and modifies sedentary lifestyles in older patients with CAD. By providing tailored health information aligned with patients’ comprehension levels and empowering them to seek information independently, healthcare providers can facilitate better disease management and mitigate physical frailty. Thus, a supportive environment provided by healthcare professionals may improve health literacy in older patients with CAD, promote physical activity, and reduce sedentary lifestyles to prevent physical frailty [39].

Moreover, healthcare professionals should recognize that improving the health literacy of older patients with CAD can help them better understand their health status, make informed decisions, and adhere to treatment regimens. Simultaneously, interventions to reduce sedentary lifestyle time and promote physical activity among older patients can slow the progression of physical frailty and improve overall quality of life. This integrated approach has the potential to address the multifaceted nature of physical frailty in older patients with CAD. Future research should focus on longitudinal designs to explore causal relationships and examine the long-term effects of interventions targeting these mediators. In addition, tailored strategies that incorporate cultural and environmental considerations are essential for effective implementation in diverse populations.

This study had several limitations. First, the ability to establish causal relationships between variables was limited by the use of a cross-sectional design with a convenience sample of older patients with CAD. Second, data collection relied on self-reported questionnaires, which may have led to an overestimation of adherence owing to perceived social desirability and recall bias. The inclusion of objective measures in future research may improve the accuracy of variable assessments. Third, because the demographic and clinical characteristics of older patients with CAD may influence physical frailty, future research should focus on replication or large-scale studies that control for these variables. However, this study did not control for these characteristics, which should be addressed in future investigations to increase the validity of the present findings. Finally, the relatively small sample size reduces the generalizability of the findings. To address these limitations, future studies should consider longitudinal designs with larger sample sizes to better identify the predictive factors of physical frailty in older patients with CAD and to provide more robust evidence.

## 5. Conclusions

The present study is among the first to examine the relationship between perceived social support and physical frailty in older Korean patients with CAD, using a dual mediation model. The results showed that perceived social support influenced physical frailty through the serial mediating effects of health literacy and a sedentary lifestyle as well as through individual mediating pathways involving each factor separately. To effectively address physical frailty in older patients with CAD and poor perceived social support, it is crucial to develop tailored interventions that focus on modifying sedentary lifestyles while incorporating strategies to improve health literacy. Future longitudinal research is needed to deepen the understanding of the mechanisms linking perceived social support and physical frailty while examining additional physical and psychosocial factors to strengthen the evidence base.

## Figures and Tables

**Table 1:** Demographic and clinical characteristics of older patients with CAD (N = 182).

Variables	Characteristic	*n* (%)	Mean ± SD
Age (years)			67.59 ± 7.49
Gender	Male	116 (63.7)	
	Female	66 (36.3)	
Education level	≤Middle school	80 (44.0)	
	High school	62 (34.1)	
	≥College	40 (22.0)	
Living arrangement	Alone	60 (33.0)	
	Family	122 (67.0)	
Time since diagnosis (years)			1.67 ± 3.56
CCS angina grade	Class I	74 (40.7)	
	Class II	55 (30.2)	
	Class III~IV	53 (29.1)	
LVEF (%)	≤40	31 (17.0)	
	41~49	93 (51.1)	
	≥50	58 (31.9)	
Comorbidity	Hypertension	126 (69.2)	
	Diabetes mellitus	66 (36.3)	
	Atrial fibrillation	31 (17.0)	
	Heart failure	38 (20.9)	

SD = standard deviation; CCS = Canadian Cardiovascular Society; LVEF = left ventricular ejection fraction.

**Table 2:** Correlations among the main variables (N = 182).

Variables		1	2	3
	*n* (%) or Mean ± SD	*r* (*p*)	*r* (*p*)	*r* (*p*)
1. PSS	75.97 ± 14.62	1		
2. HL	7.53 ± 2.99	0.47 (<0.001)	1	
3. SL(h/day)	8.08 ± 2.45	−0.54 (<0.001)	−0.49 (<0.001)	1
4. PF	127 (69.8)	−0.44 (<0.001) *	−0.41 (<0.001) *	0.55 (<0.001) *

* rpb = point-biserial correlation; r = Pearson’s correlation; SD = standard deviation; PSS = perceived social support; HL = health literacy; SL = sedentary lifestyle; PF = physical frailty.

**Table 3:** The direct effects of variables in the dual mediation model on physical frailty (N = 182).

Path	Direct Effects	B	SE	t	*p*	95% CI	Adjusted. R^2^	F (*p*)
a1	PSS → HL	0.098	0.014	7.049	<0.001	0.071~0.126	0.22	49.69 (<0.001)
a2	PSS → SL	−0.067	0.011	−5.979	<0.001	−0.089~−0.045	0.37	52.54 (<0.001)
d21	HL → SL	−0.244	0.053	−4.589	<0.001	−0.349~−0.139		
c’	PSS→ PF	−0.025	0.005	−3.008	<0.001	−0.036~−0.015	0.52	65.19 (<0.001)
b1	HL → PF	−0.088	0.024	−2.163	0.003	−0.136~−0.041		
b2	SL → PF	0.165	0.032	8.028	<0.001	0.102~0.228		

B = unstandardized beta; SE = standard error; CI = confidence interval; PSS = perceived social support; HL = health literacy; SL = sedentary lifestyle; PF = physical frailty.

**Table 4:** The bootstrap results for indirect effects on physical frailty (N = 182).

Indirect Effects	Point Estimate	Boot SE	Boot LLCI~Boot ULCI
Total indirect effects	−0.290	0.005	−0.034~−0.014
Indirect 1: PSS → HL →PF	−0.106	0.003	−0.016~−0.002
Indirect 2: PSS → SL → PF	−0.135	0.003	−0.019~−0.005
Indirect 3: PSS → HL → SL → PF	−0.048	0.001	−0.007~−0.001

SE = standard error; LLCI = lower limit confidence interval; ULCI = upper limit confidence limit interval; PSS = perceived social support; HL = health literacy; SL = sedentary lifestyle; PF = physical frailty

## Data Availability

Data sharing is not applicable to this article as the data analyzed in the current study contain participant medical information. The sharing of this information would breach patient privacy, confidentiality, and the ethics approval gained for the study.

## Abbreviations

The following abbreviations are used in this manuscript:

CAD	coronary artery disease
CCS	Canadian Cardiovascular Society
LVEF	left ventricular ejection fraction
HCCQ	Health Care Climate Questionnaire
BHLS	Brief Health Literacy Screeners
GPAQ	Global Physical Activity Questionnaire
CI	confidence interval
